# Screen time and physical activity during adolescence: longitudinal effects on obesity in young adulthood

**DOI:** 10.1186/1479-5868-4-26

**Published:** 2007-06-08

**Authors:** Janne E Boone, Penny Gordon-Larsen, Linda S Adair, Barry M Popkin

**Affiliations:** 1 Department of Nutrition, Schools of Public Health and Medicine, The University of North Carolina at Chapel Hill, Chapel Hill, NC, USA; 2 Carolina Population Center, The University of North Carolina at Chapel Hill, Chapel Hill, NC, USA

## Abstract

**Background:**

The joint impact of sedentary behavior and physical activity on obesity has not been assessed in a large cohort followed from adolescence to adulthood.

**Methods:**

Nationally representative longitudinal data from Waves II (1995; mean age: 15.9) and III (2001; mean age: 21.4) of the National Longitudinal Study of Adolescent Health (n = 9,155) were collected. Sex-stratified multivariate logistic regression analysis assessed the odds of obesity associated with Wave II MVPA and screen time, controlling for sociodemographic characteristics and change in MVPA and screen time from Wave II to III. Obesity was defined using body mass index (BMI, kg/m^2^) International Obesity Task Force cut-points at Wave II and adult cut-points at Wave III (BMI ≥ 30).

**Results:**

In males, adjusted odds of prevalent obesity was strongly predicted by MVPA bouts [OR (95% CI): OR_6 vs. 1 MVPA bouts _= 0.50 (0.40, 0.62); OR_4 vs. 40 hrs screen time _= 0.83 (0.69, 1.00)]. In females, greater MVPA bouts and lower screen time correlated with lower prevalent obesity [OR (95% CI): OR_6 vs. 1 MVPA bouts _= 0.67 (0.49, 0.91); OR_4 vs. 40 hrs screen time _= 0.67 (0.53, 0.85)]. Longitudinally, adolescent screen time hours had a stronger influence on incident obesity in females [OR (95% CI): OR_4 vs. 40 hrs _= 0.58 (0.43, 0.80)] than males [OR (95% CI): OR_4 vs. 40 hrs _= 0.78 (0.61, 0.99)]. Longitudinal activity patterns were not predictive of incident obesity.

**Conclusion:**

Reducing screen time during adolescence and into adulthood may be a promising strategy for reducing obesity incidence, especially in females.

## Background

Obesity is a major and rapidly growing global public health concern and is associated with significant morbidity and mortality [1-3]. In the past three decades, obesity has tripled among adolescents [4] and more than doubled among 20–39 year olds in the U.S. [5]. A substantial amount of weight gain [6-9], concomitant with precipitous declines in physical activity [10-13], occurs in the transition from adolescence to young adulthood.

Physical activity and sedentary behavior have become major focal areas in obesity research, interventions, and policies. Both have been linked to obesity among adolescents [14-17] and adults [18-20] in observational studies and assessed as target behaviors in randomized controlled trials among young children and adolescents [21-25], but full understanding of optimal behavior patterns for obesity prevention is far from complete. Sedentary behaviors, such as television viewing and computer games, may influence energy balance through displacement of physical activity [26], increased energy intake [27,28], or reduced metabolic rate [29]. Sedentary behaviors are generally more strongly and consistently associated with obesity than physical activity [16], and are thus commonly targeted by interventionists working with young populations.

Physical activity and sedentary behavior often co-occur [30,31] despite being inversely correlated [26,32], but there are few studies that examine their combined effects on obesity development over time. Furthermore, longitudinal effects from adolescence to adulthood are particularly important due to high risk of obesity onset and the abundance of changes in lifestyle and environment during this transition period [33,34]. Unfortunately, longitudinal studies examining physical activity and sedentary activity in relation to obesity are sparse: the only American observational cohort that captures the adolescent to adulthood transition is limited to females in three cities [17]; international studies that capture this lifecycle phase assess sedentary activity [15,35] or physical activity [36,37], but not both; and randomized controlled trials are usually of short duration (less than one year) [21-24,38] and do not continue into young adulthood [25].

In this study, we assess the combined association of moderate to vigorous physical activity (MVPA) and TV/video viewing (screen time) on incident obesity in a large nationally representative survey of adolescents followed through young adulthood. We hypothesized that cross-sectional and longitudinal patterns of physical activity would mitigate the adverse association between sedentary behavior and both current and incident obesity.

## Methods

### Study Population

The study population includes more than 20,000 individuals enrolled in The National Longitudinal Study of Adolescent Health (Add Health), a prospective cohort study of adolescents, representative of the U.S. school-based population in grades 7 to 12 in 1994–95, and followed into adulthood. Add Health included a core sample plus subsamples of selected minority and other groupings collected under protocols approved by the Institutional Review Board at the University of North Carolina at Chapel Hill. The survey design and sampling frame have been discussed elsewhere [39]. Add Health includes three waves: Wave I (1995) did not collect measured height and weight, so we used Wave II (13,570 eligible adolescents, measured April to August, 1996) and Wave III (14,322 eligible young adults, measured August 2001 and April 2002) samples in the current analysis (10,828 total eligible longitudinal respondents). Wave II followed the school-based sample of adolescents who did not graduate prior to 1996; high school dropouts and older high school students were included. Eligible respondents included those with sample weights. Females reporting pregnancy for Wave II or Wave III (n = 378) and individuals who used a walking aid (e.g., cane, crutches, or wheelchair) (n = 57); were less than 13 or greater 20 years of age at Wave II, or less than 19 or greater than 26 years of age at Wave III (n = 115); or had incomplete physical activity, sedentary behavior, height, weight, or relevant demographic data (n = 1,231) were excluded from analysis. Less than 7% of Wave II respondents were between 18 and 20 years – these were typically older adolescents who were still enrolled in school; despite being older than adolescent chronological age, they were retained in the Wave II sample: (a) to maintain consistency with the sampling design for national representation and (b) based on their lifecycle stage "of school-age" in terms of social influences and life stage. Our final sample includes 9,155 adolescents, 13–20 years of age (mean = 15.9 ± 0.12) at Wave II and 19–26 years of age (mean = 21.4 ± 0.11) at Wave III for descriptive and logistic regression analysis.

### Study Variables

Wave II and III in-home surveys of study participants provided physical activity, sedentary behavior, and body mass index [BMI: weight (kg)/height (m^2^)] data. Wave I in-home surveys of parents provided income and education data. Education was the highest level of education attained by either parent. Where missing (n = 1,502; 13.9%), income was imputed using a method similar to that used in other national surveys to deal with missing data [40,41]. Race and ethnicity were determined primarily from adolescent self-report; parent interviews were used as a secondary source.

#### Obesity

Body Mass Index (BMI) was computed from measured height and weight at Waves II and III. Adolescent respondents were classified as obese at Wave II using International Obesity Task Force (IOTF) BMI ≥ 30 kg/m^2^-equivalent age- and sex-specific BMI cut-points [42]. The IOTF cut-points provide comparability to otherwise discrepant obesity definitions for adolescents and adults [6,42], which is vital for longitudinal analysis spanning the adolescent and adult years. At Wave III, when respondents were 19 years and older, the adult obesity cut point (BMI ≥ 30 kg/m^2^) was used [43,44]. Incident obesity was defined as BMI ≥ 30 kg/m^2 ^among Wave III respondents who were not obese (BMI below the age- and sex-specific IOTF obesity cutpoint) at Wave II.

#### Physical Activity and Screen Time

Described in detail elsewhere [45,46], Add Health surveys employed a standard activity recall. While the recall has not undergone validity or reliability testing, it was based on self-report questionnaires that have been validated in other large-scale epidemiologic studies with regard to physical activity [47]. However, validation of self-reported sedentary behavior is scant [48]. In this study, physical activity was defined as weekly frequency of moderate to vigorous physical activity (MVPA; includes skating & cycling, exercise, and active sports), hereafter referred to as "bouts". Screen time was defined as hours of television and video viewing per week. The screen time distribution included a very long tail; thus, screen time greater than 80 hours per week (Wave II: 81–120 hours, n = 131, 1.2%; Wave III: 81–198 hours, n = 119, 1.1%) was truncated at 80 hours in order to stabilize regression estimates. Longitudinal measures were created as change variables, representing the change in MVPA bouts and truncated screen time hours from Wave II to Wave III.

### Statistical Analysis

Statistical analyses were performed using Stata, version 9.1 (StataCorp, College Station, TX) [49]. Descriptive analyses used post-stratification sample weights for national representation; adjusted Wald tests compared means and design-based F-tests compared distribution of categorical variables between obese and non-obese subgroups. All analyses used multiple stages of cluster sampling to adjust for survey design effects.

All analyses were stratified by sex due to known differences in physical activity and sedentary behavior patterns between males and females [12,34]. Logistic regression models were used to assess correlates of adolescent obesity status (Wave II) and predictors of obesity incidence from adolescence to young adulthood (Wave II to Wave III). Wave II obesity prevalence models included Wave II physical activity and Wave II screen time. Wave III obesity incidence models excluded respondents obese at Wave II and assessed Wave II physical activity, Wave II screen time, and physical activity & screen time change variables (Wave III-Wave II). All quadratic terms related to physical activity or screen time and all potential interactions between Wave II physical activity and screen time and change variables were assessed in each model; non-significant (p ≥ 0.10) terms were excluded. Age, race/ethnicity (White, Black, Native American, Asian, Hispanic), household income tertile, and highest parental education are established confounders in behavior-obesity relationships, so they were included in all models. Due to less consistent relationships involving season, smoking status, and geographic region, we tested these variables empirically for confounding and included them in the model as control variables if their inclusion resulted in at least a 10% change in estimate of the odds ratio for obesity related to physical activity and/or screen time.

To illustrate the effects of shifts in continuous physical activity and screen time variables on incident obesity, model estimates were used to (1) calculate odds ratios for incident obesity and (2) predict obesity incidence based on assigned physical activity and sedentary behavior profiles. Odds ratios compare selected physical activity and screen time levels to an "undesirable" referent profile of low physical activity (1 bout per week) and high screen time (40 hours per week). These values correspond to the 10^th ^and 90^th ^percentiles, respectively, at Wave II for the total sample; there was no reason to suspect differential reporting or differential associations with obesity by sex, so combined values for males and females were used to provide consistent comparisons.

Given the complexity of our models, involving quadratic terms and change variables, we used coefficients from the models along with experimentally assigned physical activity and screen time values to predict obesity incidence. We (a) fit logistic models of obesity incidence from adolescence to young adulthood (Wave II to Wave III) including Wave II physical activity, Wave II screen time, and physical activity and screen time change variables (Wave III-Wave II), (b) assigned specified physical activity-screen time combinations to all members of the cohort, then (c) applied model coefficients from (a) to the experimentally assigned physical activity and screen time values. These results provide predicted obesity incidence at specified physical activity and screen time levels, providing the opportunity for "natural" experiments involving simulated manipulations of the activity and screen time profiles and, as such, aiding in the interpretation of model results.

## Results

### Sample characteristics

A total of 4,879 males and 4,276 females met study criteria. Obesity prevalence by sex and wave of survey are presented in Table 1. A considerable proportion of males (11.5%) and (10.9%) of females were obese at Wave II, and rates approximately doubled by Wave III to 21.1% for males and 23.9% for females. Obesity incidence from Wave II to III was 13.2% (SE = 0.76) among males and 15.9% (SE = 0.96) among females.

**Table 1:** Weighted obesity prevalence^a ^[% (SE)] (Waves II and III)^b^

	MALES		FEMALES	
	
	Wave II	Wave III	Wave II	Wave III
Count	4879	4879	4276	4276
Total Sample	11.5 (0.73)	21.1 (0.94)	10.9 (0.74)	23.9 (1.18)
Parental Education				
< HS	12.0 (1.88)*	22.6 (2.57)	16.3 (1.87)**	37.0 (3.02)**
HS/GED	13.9 (1.36)*	23.2 (1.72)	11.8 (1.27)**	26.5 (1.84)**
Some College	11.7 (1.26)*	20.7 (1.31)	9.2 (1.06)**	22.0 (1.60)**
College/Grad	8.2 (1.07)*	18.3 (1.58)	8.6 (1.48)**	15.9 (1.77)**
Income Tertile				
1^st ^Tertile	13.8 (1.41)*	23.2 (1.74)**	13.3 (1.22)**	30.2 (2.12)**
2^nd ^Tertile	11.6 (0.94)*	22.5 (1.36)**	11.7 (1.09)**	25.1 (1.56)**
3^rd ^Tertile	9.2 (1.00)*	17.3 (1.39)**	7.7 (1.19)**	16.9 (1.44)**
Race				
White	11.4 (0.92)	20.4 (1.15)	10.0 (0.91)**	21.7 (1.31)**
Black	12.1 (1.58)	23.6 (2.13)	16.8 (1.68)**	35.8 (3.02)**
Native American	21.5 (10.33)	40.9 (20.19)	17.0 (6.63)**	42.2 (11.89)**
Asian	6.4 (2.38)	18.5 (3.76)	2.6 (1.25)**	9.0 (2.96)**
Hispanic	12.4 (1.89)	21.4 (2.18)	12.5 (1.70)**	28.6 (2.40)**
Region				
West	10.0 (1.57)	20.4 (2.71)	7.9 (1.27)	21.6 (2.08)*
Mid-West	11.9 (1.48)	21.6 (1.80)	12.0 (1.49)	23.5 (2.51)*
South	12.7 (1.18)	22.4 (1.51)	11.9 (1.12)	28.1 (1.84)*
Northeast	8.7 (1.21)	17.1 (1.71)	8.4 (1.81)	17.0 (2.30)*
Smoking				
Never	11.3 (0.76)	21.9 (1.29)	10.3 (0.77)	22.9 (1.46)
Former^c^	14.3 (2.64)	18.8 (1.67)	10.9 (1.64)	26.9 (2.31)
Current	11.3 (1.70)	21.2 (1.50)	13.4 (1.57)	24.2 (1.94)
Season				
Winter	--^d^	18.8 (1.52)	--^d^	21.9 (2.28)
Spring	12.9 (1.02)**	20.5 (2.04)	11.4 (1.00)	22.0 (3.18)
Summer	9.8 (0.84)**	21.9 (2.44)	10.1 (1.00)	26.5 (2.12)
Fall	--^e^	22.5 (1.27)	--^e^	24.5 (1.26)

* p < 0.05 within wave and sex; ** p < 0.01 within wave and sex

^a ^Obesity defined as BMI ≥ 30 kg/m^2 ^equivalent age- and sex-specific IOTF cut-points at Wave II; BMI ≥ 30 kg/m^2 ^at Wave III.

^b ^National Longitudinal Study of Adolescent Health [N = 9,155]. Weighted for national representation, standard errors corrected for survey design effects of multiple stage cluster sampling.

^c ^Combined with "Current" in regression modeling for Wave II

^d ^Variable is not applicable for Wave II

^e ^Insufficient cell size; combined with Summer for Wave II

At Waves II and III, obesity prevalence was similar by smoking status and, except for males at Wave II, season. Obesity prevalence was significantly higher among those with lower household income and, except for males at Wave III, lower parental education. Among females, obesity prevalence also varied by race at both waves and region at Wave III (Table 1). Non-obese females were slightly younger than obese females at both waves (Table 2).

**Table 2:** Descriptive statistics for age, physical activity, and screen time [mean (SE)] (Waves II and III)^a^

	MALES				FEMALES			
	
	Wave II		Wave III		Wave II		Wave III	
	
	Not Obese	Obese	Not Obese	Obese	Not Obese	Obese	Not Obese	Obese
Count	4529	550	3831	1048	3823	453	3289	987
Age	16.0 (0.12)	15.9 (0.19)	21.5 (0.11)	21.6 (0.14)	15.8 (0.12)**	16.0 (0.14)**	21.2 (0.12)**	21.5 (0.13)**
MVPA (bouts/wk)^b^	4.1 (0.06)**	3.7 (0.13)**	2.5 (0.04)	2.5 (0.09)	3.4 (0.07)**	2.9 (0.12)**	2.1 (0.05)	2.0 (0.08)
MVPA (change)^b^	--^c^	--^c^	-0.3 (0.01)**	-0.2 (0.02)**	--^c^	--^c^	-0.2 (0.01)	-0.1 (0.02)
Screen Time (hrs/wk)^d^	19.3 (0.45)*	21.8 (1.11)*	18.7 (0.40)	20.3 (0.82)	15.9 (0.53)**	20.3 (1.19)**	15.3 (0.39)*	21.2 (0.96)*
Screen Time (change)^d^	--^c^	--^c^	-0.7 (0.44)	-0.4 (0.97)	--^c^	--^c^	-0.2 (0.49)**	2.0 (0.98)**

* Significant difference by obesity status within wave and gender (p < 0.05). ** Significant difference by obesity status within wave and gender (p < 0.01).

^a ^National Longitudinal Study of Adolescent Health [N = 9,155]. Weighted for national representation, standard errors corrected for survey design effects of multiple stage cluster sampling

^b ^MVPA = moderate to vigorous physical activity. MVPA (change) = (Wave III MVPA) - (Wave II MVPA)

^c ^Variable is not applicable for Wave II.

^d ^Screen Time = hours/week of television and video viewing. Screen Time (change) = (Wave III Screen Time) - (Wave II Screen Time)

Descriptive statistics for physical activity and screen time by sex, wave of survey, and obesity status are presented in Table 2. At Wave II, non-obese males and females reported more physical activity and less screen time than obese males and females. Males not obese at Wave III experienced a slightly greater decline in physical activity from Wave II to Wave III than those who were obese. Females not obese at Wave III reported less screen time at Wave III and nearly no change in screen time from Wave II to Wave III as opposed to an increase in screen time among females obese at Wave III.

### *Cross-Sectional *association between physical activity & sedentary behavior and obesity during adolescence

Among males, odds of prevalent obesity during adolescence were lower with more bouts of physical activity and higher, although to a lesser degree, with more hours of screen time (Table 3). Among females, both physical activity and screen time were strong correlates of odds of obesity, although physical activity suggested a slight curvilinear relationship in which odds of obesity peaked at two to three physical activity bouts per week.

**Table 3:** Cross-sectional physical activity and screen time as predictors of obesity status of participants (Wave II)^a^

	MALES		FEMALES	
	
	coeff (95% CI)	sig	coeff (95% CI)	sig
MVPA (Wave II)^b^	-0.1382 (-0.1811, -0.0953)	<0.0001	0.1038 (-0.0439, 0.2515)	0.167
MVPA^2 ^(Wave II)^b^	--^c^		-0.0264 (-0.0493, -0.0034)	0.025
Screen Time (Wave II)^d^	0.0051 (0.0000, 0.0102)	0.049	0.0110 (0.0046, 0.0175)	0.001

^a ^National Longitudinal Study of Adolescent Health. Coefficients and 95% confidence intervals were obtained from multivariate sex-specific logistic regression models predicting Wave II obesity and accounting for sampling strategy and including all variables in the table as well as age, household income (parental), highest parental education, and race. Smoking status, season, and region did not affect model estimates so they were excluded from both models. (n_males _= 4,879, n_females _= 4,276)

^b ^MVPA = moderate to vigorous physical activity (bouts/week)

^c ^The quadratic term (MVPA^2^) was not significant for males so it was excluded from the model.

^d ^Screen Time = television and video viewing (hours/week)

#### *Longitudinal *association between adolescent physical activity & sedentary behavior and incident obesity between adolescence and early adulthood Coefficients for incident obesity from adolescence to adulthood

For both males and females, significantly higher odds for incident obesity were observed with greater hours of screen time during adolescence as well as an increase in screen time from adolescence to early adulthood (Table 4). Neither physical activity during adolescence nor change in physical activity significantly predicted incident obesity in males. Similar to the cross-sectional analysis, there was a slight curvilinear relationship for physical activity among females.

**Table 4:** Physical activity and screen time as predictors of 5-year incident obesity (Wave II to III)^a^

	MALES		FEMALES	
	
	coeff (95% CI)	sig	coeff (95% CI)	sig
MVPA (Wave II)^b^	-0.0064 (-0.0620, 0.0492)	0.821	0.1583 (-0.0147, 0.3313)	0.073
MVPA^2 ^(Wave II)^b^	--^c^		-0.0242 (-0.0477, -0.0008)	0.043
MVPA (change)^b^	0.0094 (-0.0348, 0.0535)	0.675	-0.0174 (-0.0686, 0.0338)	0.503
Screen Time (Wave II)^d^	0.0070 (0.0002, 0.0139)	0.043	0.0150 (0.0062, 0.0237)	0.001
Screen Time (change)^d^	0.0062 (0.0009, 0.0115)	0.021	0.0119 (0.0051, 0.0186)	0.001

^a ^National Longitudinal Study of Adolescent Health. Coefficients and 95% confidence intervals were obtained from multivariate gender-specific logistic regression models predicting incident obesity from Wave II to Wave III, and accounting for sampling strategy and including all variables in the table as well as age at Wave III, household income (parental), highest parental education, race, smoking status at Wave III (females only), and season at Wave III (males only). Region did not affect model estimates so it was excluded from both models. (n_males _= 4,329, n_females _= 3,823)

^b ^MVPA = moderate to vigorous physical activity (bouts/week). MVPA (difference) = (MVPA at Wave III) - (MVPA at Wave II)

^c ^The quadratic term (MVPA^2^) was not significant for males so it was excluded from the model.

^d ^Screen Time = television and video viewing (hours/week). Screen Time (change) = (Screen Time at Wave III) - (Screen Time at Wave II)

#### Relative odds of incident obesity from adolescence to young adulthood

Based on coefficients from the model displayed in Table 4, Table 5 presents odds ratios for Wave III incident obesity at selected levels (10^th^, 25^th^, 50^th^, 75^th^, 90^th ^percentiles) of Wave II physical activity & screen time for males and females, respectively (referent profile: 1 bout of physical activity and 40 hours screen time; all other variables were held constant). Consistent with the model estimates, relative odds of incident obesity become more protective with lower adolescent screen time regardless of physical activity. This association was more dramatic in females than in males: for example, very low screen time (4 hours per week) reduced relative odds of incident obesity by over 40% (OR_4 hours, 1 bout _= 0.58) for females and over 20% for males (OR_4 hours, 1 bouts _= 0.78) with very low physical activity (1 bout MVPA per week). These patterns were similar with negative, constant, and positive temporal shifts in screen time. Furthermore, this pattern is nearly identical at each level of physical activity, despite statistical significance in the logistic regression model for females.

**Table 5:** Physical activity and screen time odds ratios for 5-year incident obesity (Wave II to III)^a^

MVPA^b ^(bouts/wk – Wave II)					
Screen Time^c^	1	2	3	5	6
(hrs/wk – Wave II)	MALES [OR (95% CI)]				
					
4	0.78 (0.61, 0.99)*	0.77 (0.59, 1.00)	0.77 (0.57, 1.03)	0.76 (0.52, 1.09)	0.75 (0.50, 1.14)
7	0.79 (0.63, 0.99)*	0.79 (0.62, 1.01)	0.78 (0.60, 1.03)	0.77 (0.54, 1.10)	0.77 (0.52, 1.14)
13	0.83 (0.69, 0.99)*	0.82 (0.67, 1.01)	0.82 (0.64, 1.03)	0.81 (0.58, 1.11)	0.80 (0.55, 1.16)
25	0.90 (0.81, 1.00)	0.89 (0.79, 1.02)	0.89 (0.75, 1.05)	0.88 (0.67, 1.14)	0.87 (0.63, 1.20)
40	1.00^d^	0.99 (0.94, 1.05)	0.99 (0.88, 1.10)	0.97 (0.78, 1.22)	0.97 (0.73, 1.28)
					
	FEMALES [OR (95% CI)]				
					
4	0.58 (0.43, 0.80)*	0.64 (0.46, 0.89)*	0.66 (0.46, 0.95)*	0.61 (0.42, 0.91)*	0.55 (0.36, 0.84)*
7	0.61 (0.46, 0.81)*	0.66 (0.49, 0.90)*	0.69 (0.49, 0.97)*	0.64 (0.44, 0.93)*	0.58 (0.39, 0.86)*
13	0.67 (0.53, 0.84)*	0.73 (0.56, 0.94)*	0.75 (0.56, 1.01)	0.70 (0.50, 0.99)	0.63 (0.44, 0.91)*
25	0.80 (0.70, 0.91)*	0.87 (0.73, 1.03)	0.90 (0.72, 1.13)	0.84 (0.63, 1.12)	0.75 (0.54, 1.05)
40	1.00^d^	1.09 (0.98, 1.22)	1.13 (0.94, 1.36)	1.05 (0.81, 1.37)	0.95 (0.69, 1.30)

* OR is significantly different than 1.0 (p < 0.05).

^a ^National Longitudinal Study of Adolescent Health. Odds ratios and 95% confidence intervals from multivariate gender-specific logistic regression models (shown in Table 4; n_males _= 4,329, n_females _= 3,823) predicting incident obesity from Wave II to Wave III, and accounting for sampling strategy and including all variables in the table as well as age at wave III, household income (parental), highest parental education, race, smoking status at Wave III (females only), and season at Wave III (males only).

^b^Selected MVPA (moderate to vigorous physical activity) levels correspond to the 10^th^, 25^th^, 50^th^, 75^th^, and 90^th ^percentiles for males and females combined during Wave II.

^c ^Selected Screen Time (television and video viewing) levels correspond to the 10^th^, 25^th^, 50^th^, 75^th^, and 90^th ^percentiles for males and females combined during Wave II.

^d ^Reference profile is 1 bout MVPA, 40 hours Screen Time. All other variables are held constant.

#### Adjusted obesity incidence from adolescence to young adulthood

**Figure 1:**
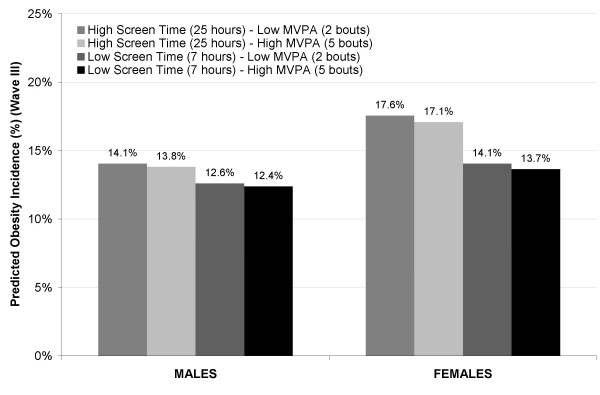
**Predicted obesity incidence (%) based on obesity incidence models (Wave II to III)^a^**. ^a ^National Longitudinal Study of Adolescent Health. Predicted obesity incidence (%) based on multivariate sex-specific logistic regression models (shown in Table 3) predicting incident obesity from Wave II to Wave III, and accounting for sampling strategy and including baseline MVPA (moderate to vigorous physical activity, bouts/week, Wave II) and screen time (television and video viewing, hours/week, Wave II), as well as age at wave III, household income, highest parental education, race, and season at Wave III. Selected MVPA and Screen Time levels correspond to the 25^th ^and 75^th^percentiles for males and females combined during Wave II. Assumes no change in screen time.

Also based on model coefficients displayed in Table 4, Figure 1 shows predicted obesity incidence at experimentally assigned combinations of physical activity and screen time profiles (25^th ^and 75^th ^percentiles of MVPA and screen time). Change in MVPA from Wave II to III was held constant due to lack of statistical significance as shown in Table 4, and change in screen time was not displayed because patterns were similar across the relatively narrow range of observed values.

For both males and females, predicted obesity incidence was highest for high screen time profiles during adolescence and the effects of experimental changes in physical activity were minimal, although the effects of experimental changes in screen time were more pronounced for females than males. Given constant screen time, predicted obesity incidence for males with low screen time (7 hours/week) paired with high MVPA (5 bouts/week) during adolescence was 12.4%, versus 14.1% for a less desirable profile of high screen time (40 hours/week) and low MVPA (1 bout/week). In comparison, analogous predicted obesity incidences for females were 13.7% and 17.6% (Figure 1). Experimental profiles involving changes in screen time produced similar patterns: reductions in screen time from adolescence to adulthood predicted lower obesity incidence, but not enough to overcome the obesity-promoting associations of screen time during adolescence.

## Discussion

In this large, nationally representative, longitudinal cohort, weekly hours of screen time during adolescence independently and significantly predicted incident obesity in early adulthood. Fewer weekly hours of screen time during adolescence reduced the relative odds of incident obesity by over 40% among females and over 20% among males. Contrary to our hypothesis, longitudinal patterns of physical activity were less important predictors of incident obesity. Our results suggest that physical activity, if not maintained, may not be protective in the long-term and may not counteract the negative effects of screen time during adolescence and over time. Declines in screen time during the transition from adolescence to young adulthood were associated with lower obesity incidence, particularly for females, but not enough to overcome the obesity-promoting effects of screen time during adolescence. Our longitudinal results, including stronger results for females [35], are consistent with other studies summarized in a recent literature review [16] and in other recent studies showing a positive relationship between sedentary behavior and body weight [15,16,50] and no longitudinal relationship between physical activity and obesity [36,37], although existing literature is inconsistent.

In contrast, our cross sectional analysis suggests a strong protective association between physical activity and prevalent obesity for adolescent males, while both physical activity and screen time were important factors for adolescent females. This protective cross-sectional association between physical activity and obesity is not observed longitudinally, perhaps because such associations are diminished by other important lifestyle changes occurring during this lifecycle period [51]. Gordon-Larsen et al. showed a positive relationship between physical activity and incident obesity among adolescents between Waves I and II of Add Health [52], which is consistent with this explanation because it studied a one year follow-up period in contrast with the five year period assessed in the current study. Additionally, as noted in the Must and Tybor review [16], cross-sectional associations may reflect causal effects of physical activity patterns on obesity, but they could also reflect limited capacity to engage in exercise or sports due to extreme obesity, lack of social support for overweight or obese adolescents, or weight loss efforts.

In females, the likelihood of obesity peaked at two to three MVPA bouts per week, perhaps reflecting reverse causality as described above or inaccurate reporting of MVPA. Those engaging in two or three bouts of MVPA may have been more likely to exercise for weight loss or maintenance and hence at greater risk of obesity. Alternatively, reporting two or three bouts may reflect over-reporting to a greater extent than those with high MVPA frequency; that is, due to social desirability, those with no MVPA may over-report within a reasonable range, resulting in high obesity prevalence and incidence in this MVPA range.

For females, reduced screen time was associated with a greater relative reduction of a larger absolute obesity incidence than for males (13% for males versus 16% for females), resulting in a greater potential impact of screen time on obesity incidence. Assuming no change in screen time, experimentally assigning low versus high screen time led to predicted obesity incidence of approximately 14% and 18%, respectively (a 3 to 4 percentage point difference) for females and 12% and 14% (a 1 to 2 percentage point difference) for males. These results underscore the potential public health impact of reducing screen time during adolescence on controlling obesity incidence in females in particular.

The association between screen time and incident obesity may differ by sex due to biologic differences in changes in energy expenditure in response to screen time, in the effects of other lifestyle factors, misclassification biases by sex, or, more likely, a combination of all three. Biologic differences such as differential reductions in metabolic rate while engaging in sedentary activity are not evident in clinical research, but these studies are often limited to young children or include only one sex [29,53]. Other lifestyle factors such as changes in energy intake related to television viewing [27,28] or concurrent activities while watching television or videos could also differ between males and females. Similarly, as hypothesized by Dunstan et al. [54], screen time may be an indicator of sedentary behaviors in general, including activities such as computer use or reading, to a greater extent in females than males. Unfortunately, we are not able to quantify energy intake or overall energy expenditure in Add Health, so we were unable to investigate this issue. Recent studies in adult populations have demonstrated stronger associations between television viewing and metabolic risk factors in women than men [54-56], suggesting that this difference is not spurious and reflects important sex differences in the influence of screen time on obesity and obesity-related conditions. In addition, findings point to questions regarding assessment of screen time.

Regardless of the mechanism or whether it is a direct relationship – increased "junk food" consumption in response to advertisements, deflated metabolic rate, or some other factor – these results suggest that adolescent females who watch less television and videos during adolescence and/or reduce their viewing time from adolescence to young adulthood are less likely to develop obesity as they become young adults, regardless of their physical activity level. Thus, reducing screen time during adolescence is likely an essential component of obesity prevention, particularly for females. Furthermore, the level of screen time at which obesity odds were reduced in this study are consistent with the American Academy of Pediatrics recommendation [57] of 14 or fewer hours of television viewing per week in pediatric populations.

There are some limitations of the current study. As noted above, we could not assess energy intake, and activity data are subject to self-report bias. Misclassification is probably most dramatic among obese respondents reporting more socially desirable behavior, which would have attenuated the association between screen time and physical activity with obesity. For the same reason, those becoming obese between Waves II and III may have underreported screen time and/or over-reported physical activity to a greater extent at Wave III; this differential misclassification would have also attenuated the observed association between changes in these behaviors and Wave III incident obesity.

Second, the activity recall may not have captured all MVPA performed by respondents, and the "bouts" of activity classification is imprecise because each bout could be of any duration. Both of these factors would be expected to reduce precision and further dilute the apparent influence of physical activity. Furthermore, MVPA bouts may more accurately reflect physical activity level during adolescence, when more easily recalled organized sports practices and competitions are more common; this bias could potentially explain the lack of association between physical activity in young adulthood as well as stronger association with screen time, which may be more accurately reported, than physical activity. Finally, unmeasured factors related to both screen time and obesity such as an awareness of healthful behaviors could have contributed to these findings.

## Conclusion

Physical activity and sedentary behavior are common targets for obesity prevention and treatment, and this study assesses the extent to which they predict obesity during a critical period in which risk for obesity onset is high and adult lifestyle takes shape. Our findings support public health strategies for reducing obesity incidence in this period that focus on reducing screen time during adolescence and maintaining these screen time reductions into young adulthood. Our research further suggests that physical activity during adolescence remains important for the establishment of lifelong habits as well as short-term weight maintenance, but that physical activity levels would need to be increased substantially and sustained into adulthood in order to counteract the negative effects of screen time. Findings were complex for males and suggest that identification of possible intervention points for males is an important next step. Further investigation of the mechanism through which screen time might influence development of obesity will help to guide potential intervention strategies. Finally, additional randomized trials that assess the combined effects of sedentary behavior and physical activity on obesity over a long follow-up period that captures the transition from adolescence to adulthood might help to confirm or refute the causal nature of these relationships.

## Competing interests

There were no potential or real conflicts of financial or personal interest with the financial sponsors of the scientific project.

## Authors' contributions

JB, PG-L, LSA, and BMP contributed to the study design, JB and PG-L contributed to the data analysis. All four authors contributed to the writing and approval of the manuscript.
